# Into the Labyrinth of the Lipocalin α1-Acid Glycoprotein

**DOI:** 10.3389/fphys.2021.686251

**Published:** 2021-06-08

**Authors:** Mario Ruiz

**Affiliations:** Department of Chemistry and Molecular Biology, University of Gothenburg, Gothenburg, Sweden

**Keywords:** α1-acid glycoprotein, orosomucoid, inflammation, metabolism, ligand-binding, Lipocalin

## Abstract

α_1_-acid glycoprotein (AGP), also known as Orosomucoid (ORM), belongs to the Lipocalin protein family and it is well-known for being a positive acute-phase protein. AGP is mostly found in plasma, with the liver as main contributor, but it is also expressed in other tissues such as the brain or the adipose tissue. Despite the vast literature on AGP, the physiological functions of the protein remain to be elucidated. A large number of activities mostly related to protection and immune system modulation have been described. Recently created AGP-knockout models have suggested novel physiological roles of AGP, including regulation of metabolism. AGP has an outstanding ability to efficiently bind endogenous and exogenous small molecules that together with the complex and variable glycosylation patterns, determine AGP functions. This review summarizes and discusses the recent findings on AGP structure (including glycans), ligand-binding ability, regulation, and physiological functions of AGP. Moreover, this review explores possible molecular and functional connections between AGP and other members of the Lipocalin protein family.

## Introduction

α_1_-acid glycoprotein (AGP), also known as Orosomucoid (ORM), is member of the Lipocalin protein family and well-known for being a positive acute-phase protein. Approximately 70 years have passed since the discovery of AGP (Schmid, 1950; Weimer et al., 1950) and thousands of studies have been performed since then. In the big picture, AGP is commonly defined as a transport protein in plasma whose main function is to modulate the immune system, including cytokine secretion (Hochepied et al., 2003). Numerous *in vitro* and *in vivo* activities such as the inhibition of platelet aggregation (Costello et al., 1979), modulation of cell proliferation/differentiation (Chiu et al., 1977; Qin et al., 2017; Lee et al., 2021; Shi et al., 2021), and drug transport have been reported (Israili and Dayton, 2001). However, the exact molecular mechanism of AGP function remains to be elucidated. AGP is mostly found in plasma from hepatic origin, but other tissues/cells such as the adipose tissue, the nervous system, endothelial cells, and immune cells also express AGP, especially during inflammatory conditions (Dente et al., 1988; Sorensson et al., 1999; Hochepied et al., 2003). Indeed, a large number of pathological conditions (including many types of cancers, infection, obesity, and cardiovascular diseases) raise AGP levels in plasma (Israili and Dayton, 2001).

This work represents an overview of the multiple faces of AGP and focuses on its physiological roles. Furthermore, this review explores possible molecular and functional connections between AGP and other members of the Lipocalin protein family (summarized in Table 1).

**TABLE 1:** Summary of the associations/common features of AGP and other members of the Lipocalin protein family discussed in this mini-review. Note many other examples might exit.

Other Lipocalins	Topic discussed	Section
Several	Immunocalin group	AGP, Definition, and Molecular Characteristics
ApoD and ApoM	Progesterone binding	Endogenous Ligands
ApoM	PAF, barrier permeability	Endogenous Ligands
Lipocalin 1 and 2	Siderophore binding	*In vivo* Approaches, Transgenesis, and Knockouts
ApoD	Leptin Receptor	*In vivo* Approaches, Transgenesis, and Knockouts
RBP4 and several others	Retinol Binding	*In vivo* Approaches, Transgenesis, and Knockouts
ApoD and Lcn2	Lipocalins and Astrocyte responses	*In vivo* Approaches, Transgenesis, and Knockouts

## AGP, Definition, and Molecular Characteristics

Human AGP (hAGP) is actually not a single unique protein. Instead, two main forms of AGP coexists in humans. They are encoded by a cluster of genes: AGP1 is encoded by the *ORM1* gene and AGP2 by the *ORM2* gene. Both genes have identical structures with 5 introns (Sanchez et al., 2003), and AGP1/2 sequences only differ in 22 amino acids. Besides this complexity, *ORM1* gene has three common variants: F1, F2, and S (collectively referred as F^∗^S). Equally, AGP*2* is sometimes referred as variant A. Interestingly, other mammals have a different number of *Orm* genes. For instance, AGP is coded by three *Orm* genes in mouse, whereas rats have a single gene. The different number of “AGP-genes” in laboratory animal models could be turned into a research advantage, though this has not been much exploited yet.

**FIGURE 1:**
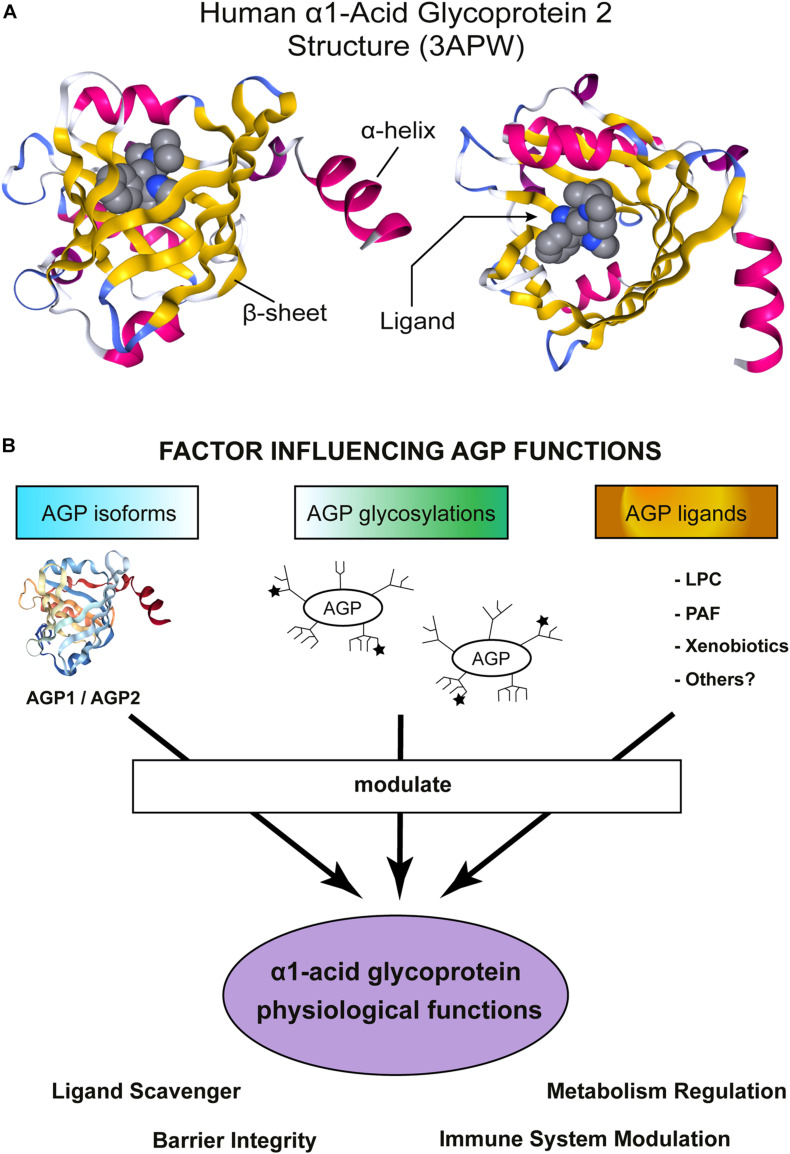
α1-Acid Glycoprotein (AGP) structure and functions. **(A)** Schematic representation of hAGP2 structure in complex with a drug in the Lipocalin cavity. In this case the ligand is the antiarrhythmic medication disopyramide (3APW) [originaly published by Nishi et al. (2011)]. The left model is a lateral view of AGP2 and the right model show the view from the top, the open side of AGP β-barrel. The secondary structures are colored: β-sheets in yellow and α-helix in magenta. The ligand (disopyramide) is represented in a bulky format to highlight the cavity inside of AGP2. The figure was generating using NGL viewer (Rose et al., 2018). **(B)** Schematic representation showing important factors influencing AGP functions. AGP is highly heterogenic and numerous activities *in vitro* and *in vivo* have been reported. AGP is an acute-phase protein that modulates the immune system and, recently, AGP has also been shown to regulate the metabolism. AGP tissue/cell type of expression determines which isoform is expressed: AGP1 or AGP2. Then, each tissue counts with a specific set of enzymes to glycosylate proteins and, indeed, multiple glycosylation patterns have been detected in AGP. The ★ symbols represent the possible presence of sLe^*x*^ groups. Note that the schematic cartoon of the glycosylation does not represent the actual complex and heterogeneous AGP glycosylation pattern. More detailed information can be found in Baerenfaenger and Meyer (2018), Keser et al. (2021). Finally, the environment where AGP is secreted allows to bind/scavenger a different set of ligands, Indeed, an enormous number of compounds are efficiently bound by AGP. The sum of all the above-mentioned factors would contribute to AGP physiological functions.

The crystals of unglycosylated AGP1/2 (produced in *E. coli*) revealed a typical Lipocalin fold comprising an eight-stranded β-barrel which is flanked by a C-terminal α-helix (Figure 1A; Schonfeld et al., 2008; Nishi et al., 2011). Four loops connect the β-sheets and a tryptophan is buried inside of the cavity. This is one of the few, but key, amino acid mostly conserved across the Lipocalin family (Flower et al., 2000; Ganfornina et al., 2000; Schiefner and Skerra, 2015). AGP2 ligand-binding region is narrower than that in AGP1, explaining the different compound-binding affinities reported for AGP1/2 (several examples can be found in Herve et al., 1993; Herve et al., 1996, 1998; Kuroda et al., 2003; Zsila et al., 2008; Nishi et al., 2011). Two disulfide bridges stabilize the structure of hAGPs (Schonfeld et al., 2008; Nishi et al., 2011). Interestingly, hAGP2 has an extra free Cys that could form a covalent binding with other proteins or participate in redox reactions, though this is merely a speculation.

α_1_-acid glycoprotein is heavily glycosylated, five N-linked glycans are present in hAGPs. These glycans represent around 45% of the AGP molecular weight and contain a high proportion of sialic acid, giving AGP its characteristic acid isoelectric point (p*I* = 2.7–3.2). In contrast, the unglycosylated AGP p*I* was calculated to be 4.97. Glycosylation increases AGP solubility but, importantly, increases its molecular weight such that it escapes glomerular filtration in the kidneys. AGP glycosylation pattern is rather complex and heterogeneous [reviewed in detail by Fournier et al. (2000); and further studied by Fernandes et al., 2015]. Multiple glycan combinations have been detected in the plasma of healthy humans (Treuheit et al., 1992; Ongay and Neususs, 2010; Baerenfaenger and Meyer, 2018; Keser et al., 2021) and changes under pathological states have been reported (De Graaf et al., 1993; Liang et al., 2019; Keser et al., 2021). More specifically, branches with sialic acid are also fucosilated creating highly biologically active sialyl-Lewis X epitopes (sLe^*x*^). It is not fully clear how glycosylation affects AGP binding toward endogenous ligands, but the fact that Asn-75 localizes near to the entrance of the binding pocket should be considered (Nishi et al., 2011). Additionally, there are documented examples where branching and fucosylation do limit drug-binding affinity (i.e., Wu et al., 2018).

Evolutionary, Lipocalins were classified in fourteen clades (I-XIV), where AGP clusters among the modern ones and included in clade XII (Gutierrez et al., 2000). AGP is only found in vertebrates and classified as an “outlier Lipocalin” because it contains only one of the three Lipocalin structurally conserved regions (SCRs) (Flower et al., 2000). Besides the traditional way of classifying Lipocalins, the term Immunocalins was proposed some time ago (Logdberg and Wester, 2000). Immunocalins would be a group of proteins sharing the Lipocalin fold and involved in immune system regulation. The founder group included: AGP, α_1_-Microglobulin, Glycodelin, and Lipocalin 2 (Lcn2 and also known as Siderocalin, NGAL, or 24p3), Complement Factor 8, γ-subunit, Tear Lipocalin (also known as Lcn1 or Von Ebner’s gland protein) and Lipocalin Prostaglandin D Synthase (L-PGDS). Even though the concept is still valid, we now know that many other Lipocalins modulate the immune response. Examples include Apolipoprotein D (ApoD) (Dassati et al., 2014) and Apolipoprotein M (ApoM) (Frej et al., 2017; Ruiz et al., 2017). Altogether then, there is a mounting evidence that the first ancestral Lipocalin may have had general defensive functions.

## AGP Ligands and Receptors

A powerful approach to investigate a Lipocalin’s physiological function(s) is often to identify its ligand(s). Such an approach would be suitable for some Lipocalins, such as RBP (Retinol Binding Protein 4), L-PGDS or ApoM. However, AGP is much more complex: it has a promiscuous ligand-binding behavior and is capable of binding hundreds of molecules from endogenous or exogenous origin. AGP has one primary high-affinity binding-site -“the classical Lipocalin binding-site”- but other sites with different capacities and lower affinity exists. Binding data for more than 300 drugs and endogenous substances were compiled several years ago and the list of compounds keeps growing (Israili and Dayton, 2001; Wishart et al., 2006; Zsila et al., 2006; di Masi et al., 2016; Smith and Waters, 2018). AGP binds mainly to basic molecules, given its highly acidic nature, but it is also able to bind neutral and acidic drugs (Taguchi et al., 2013).

### Endogenous Ligands

Several endogenous molecules bind to AGP. Catecholamines are long-known but are low-affinity ligands of AGP (Sager et al., 1987). Similarly, the ability of AGP to bind progesterone with low affinity was documented earlier (Westphal et al., 1961; Ganguly et al., 1967), and further confirmed with modern methods (Albani, 1997; Ojala et al., 2006; Huang and Hudgens, 2013). It was suggested that progesterone sequestration by AGP would represent a buffer system (Westphal et al., 1961), but not much have been experimentally demonstrated. Curiously, plasma AGP is not the only Lipocalin able to bind progesterone. ApoD was isolated as a progesterone-binding protein from mammary cystic fluid (Pearlman et al., 1973; Rassart et al., 2000) and even the complex crystalized (Eichinger et al., 2007). Additionally, recombinant ApoM showed certain ability to interact with progesterone *in vitro* (Ahnstrom et al., 2007), but no evidences of progesterone being an endogenous ApoM ligand have been presented so far.

Another report strongly argued for biliverdin as the endogenous ligand of hAGP1. Even though convincing *in vitro* data supports the binding, no evidences of biliverdin as endogenous ligand were presented (Zsila and Mady, 2008). The authors speculated that inside of the β -barrel, biliverdin might be transiently protected from enzymatic oxidation, thereby preventing accumulation of toxic bilirubin (Zsila and Mady, 2008).

In the search of physiological AGP ligand(s), a big effort was implement in Finland a few years ago (Ojala et al., 2006). Large amounts of AGP were isolated from plasma, followed by lipid extraction and mass spectrometry analysis. Significant amounts of lysophospholipids, and more specifically lysophosphatidylcholines (LPC) with unsaturated acyl chains, were identified. Further *in vitro* ligand-binding assays confirmed the highest affinity for LPC20:4, LPC18:3, and LPC18:1. However, AGP was also able to efficiently bind free fatty acids and platelet activated factor [(PAF), 1-alkyl-2-acetyl-sn-glycero-3-phosphocholine, (as also previously reported in McNamara et al., 1986)]. Finally, to highlight the biological relevance of AGP-LPC and AGP-PAF complexes, it was shown that AGP prevented LPC-induced priming and PAF-induced activation of human granulocytes (Ojala et al., 2006). Systemically, several studies have shown that AGP contributes to maintain cellular barriers in the kidneys, lungs, brain and vessels (i.e., Haraldsson et al., 1992; Johnsson and Haraldsson, 1993; Muchitsch et al., 1996, 1998, 2000; Sorensson et al., 1999) whereas LPC and PAF induce permeability (Huang et al., 2005; Hudry-Clergeon et al., 2005); suggesting that AGP could be a LPC/PAF scavenger. Interestingly, the expression of another Lipocalin, ApoM, is induced by PAF (Xu et al., 2002) and ApoM is fundamental to maintain barrier function (Christoffersen et al., 2011; Ruiz et al., 2017; Mathiesen Janiurek et al., 2019). In conclusion, the work by Ojala et al. (2006) could potentially explain the anti-inflammatory/protective effects of AGP and it probably has been the best attempt to explain the physiological relevance of AGP ligand(s).

### Xenobiotics-Binding

Many compounds show potential therapeutic capacity when examined *in vitro* or in animal models. However, the impressive ability of AGP to bind drugs sometimes represents a limitation for their clinical use (as discussed on Smith and Waters, 2018; Bteich, 2019; Bteich et al., 2021). UCN-01 (7-hydroxystaurosporine) is an anti-cancer drug and its sequestration by AGP is a classic example of AGP affecting drug pharmacokinetics and pharmacodynamics. hAGP displays an unexpected high affinity for UCN-01 and hence increases drug plasma concentration, while blocking its distribution and elimination. In contrast, canine AGP has lower affinity for UCN-01 and therefore has little effect on the pharmacodynamics of UCN-01 and rat AGP exhibited only weak and nonspecific binding to UCN-01 (Fuse et al., 1998, 1999). Interestingly, encapsulation of UCN-01 in liposomes has been proposed to reduce the impact of AGP on UCN-01 pharmacodynamics (Yamauchi et al., 2005, 2008).

Examples of recent studies on drugs bound by AGP include: Warfarin (Hanada, 2017), Pinometostat (Smith et al., 2017), Aripiprazole (Nishi et al., 2019), Imatinib (Mic et al., 2020), Voriconazole (Yuan et al., 2020), ONO-2160 (Kono et al., 2019, 2021), SCO-272 (Ebihara et al., 2018, 2019), and Brigatinib (Wang et al., 2020).

### AGP Receptors

The paragraphs above discussed the binding capabilities of AGP. But it is unclear if AGP is a passive scavenger protein or, alternatively, whether AGP delivers its cargos to particular receptors. Several membrane proteins have been reported to interact with AGP. For instance, AGP binds to the C-C chemokine receptor type 5 (CCR5) in the plasma membrane of macrophages and skeletal muscle cells (Atemezem et al., 2001; Lei et al., 2016). Both, AGP polypeptide and glycans are important for AGP-CCR5 interaction. Speculatively, the authors suggested that AGP-CCR5 association could block the infection of macrophages by the HIV-1 virus (Seddiki et al., 1997).

The presence of numerous sLe^*x*^ groups in AGP have led to depict AGP as an interacting partner of the endothelial adhesion molecules P-selectin and E-selectin. In this way, AGP binding would block the adhesion of circulating leukocytes to the endothelium upon inflammatory stimuli (Jorgensen et al., 1998; Hochepied et al., 2000). A structural model of AGP and P-selectin interaction have been calculated *in silico* (Fernandes et al., 2015). Furthermore, AGP sLe^*x*^ groups mediate the interaction of AGP with immunoglobulin-like lectins (Siglecs) (Gunnarsson et al., 2007) and modulate reactive oxygen species (ROS) generation in neutrophils (Gunnarsson et al., 2010).

Finally, the liver-expressed asialoglycoprotein receptor binds molecules of AGP in which the terminal sialic groups are missing and efficiently clears asialo-AGP from circulation (Kindberg et al., 1990; Matsumoto et al., 2010). It has been suggested that another, yet unknown, receptor would mediate AGP uptake (with sialic acid residues) (Matsumoto et al., 2010; Taguchi et al., 2013). However, it is not known if AGP is simply targeted for degradation or has also intracellular functions.

Remarkably, AGP binds to membranes and undergoes a pH-induced conformational change (a unique transition from a β-sheet-rich structure to an α-helix-rich structure) which caused a decrease in AGP affinity for progesterone (Nishi et al., 2002, 2004, 2006). This has been interpreted as a mechanism to release molecules inside of the cell (illustrated in Taguchi et al., 2013). However, this has also been interpreted in the opposite way: as a mechanism to sequester LPC/PAF from the plasma membranes where they are generated (Ojala et al., 2006). Interestingly, this unique β-sheet to α-helix transition has also been reported for Tear Lipocalin (Gasymov et al., 1998). Follow-up investigations to address the relevance of AGP β-sheet to α-helix transition *in vivo* would be highly valuable.

## *In vivo* Approaches, Transgenesis, and Knockouts

α_1_-acid glycoprotein being an acute-phase protein, most of the early *in vivo* studies were related to inflammatory insults. For that, different transgenic mouse models were initially created to study AGP. Bacterial lipopolysaccharide (LPS) strongly induced expression and liver secretion of hAGP1 in mice carrying the whole hAGP gene cluster (*ORM1*, *ORM2*, and *ORM3* genes) or a fragment with only the *ORM1* gene (Dente et al., 1988). Later, another transgenic mouse in which the rat *Orm* gene was over-expressed was made. LPS, IL-1, IL-6, or glucocorticoids were used to trigger the inflammatory response and this boosted rated AGP expression several folds (Dewey et al., 1990). In general, AGP has shown to be protective *in vivo* against inflammatory insults (as summarized in Hochepied et al., 2003). One example is that the intraperitoneal injection of hAGP (but also rat and bovine) protected against lethal shock induced by TNFα (Libert et al., 1994). However, the overexpression of rat AGP led to a more aggressive development of acute colitis (Hochepied et al., 2002).

Another example of a protective effect is that pre-administration of exogenous bovine or hAGP or transgenic over-expression of rat AGP in mice, significantly increased survival against a lethal infection with the Gram-negative bacteria *Klebsiella pneumoniae* (Hochepied et al., 2000) or *Bacillus anthracis* (Shemyakin et al., 2005). However, the molecular mechanism involved is unknown. One explanation could be the reported capacity of AGP to form complexes with LPS (Moore et al., 1997). However, AGP-LPS complexes cannot explain the documented protection against the Gram-positive *Bacillus anthracis*. Alternatively, a recent paper proposed a direct action of AGP on bacterial growth. Siderophores are small molecules secreted by bacteria to secure their iron supply (a scarce and essential micronutrient) and growth. The authors reasoned that many bacteria secrete stealth siderophores that escape Lcn2 recognition (the archetype Siderocalin), and suggested that AGP may be a “Siderocalin” and hence able to bind siderophores (Samsonov et al., 2021). Interestingly, Tear Lipocalin, a highly abundant Lipocalin in secretions, interferes with microbial growth by scavenging of siderophores (Fluckinger et al., 2004), Tear Lipocalin is, however, not present in plasma. Given the similarities between Lcn2, Tear Lipocalin and AGP, one may speculate that AGP can also neutralize siderophores escaping Lcn2 entrapment and inhibit *K. pneumoniae* or *B. anthracis* growth. Unfortunately, AGP computational experiments were inconclusive for *K. pneumoniae* siderophores (Samsonov et al., 2021). However, the results were more positive about petrobactin (one of the siderophores secreted by *B. anthracis*) being a candidate ligand for AGP (Samsonov et al., 2021). In any case, the ability of AGP to bind siderophores and inhibit bacterial growth needs to be experimentally demonstrated.

AGP1 is highly abundant in plasma (∼0.075 g/dl; ∼15 μM) (Kremer et al., 1988; Gannon et al., 2019; McDonald et al., 2020), easy to purify and represents a relatively affordable commercial source of AGP protein for *in vitro* and *in vivo* experiments. However, there are some limitations that can complicate the interpretations of the results. First, different batches of protein come from different donors and likely have distinct glycosylation patterns. Importantly, mouse and human livers possess a different set of fucosyltransferases and hAGP produced in mice lacks sLe^*x*^ (Havenaar et al., 1998). sLe^x^ groups can be important to efficiently modulate hAGP function. Thus, hAGP might be not fully functional in murine experimental models. Additionally, isolated AGP will likely come with uncharacterized ligand(s) in its cavity and variations in the purification protocols may impact their presence and nature.

The absence of AGP-KO animal model was a strong limitation to understand AGP functions, until, for the first time, an Orm1-KO mouse was published in 2016 (Lei et al., 2016). The newly created mouse mutants were first used to demonstrate that AGP1 binds to CCR5 on skeletal muscle cells to increase muscle endurance (Lei et al., 2016). Later, the same group showed that AGP1-CCR5 increased the activity of glycogen synthase (the rate-limiting enzyme in the glycogen synthesis pathway) via AMPKα2 (Qin et al., 2016). Further, they identified estrogens (as a negative) and erythromycin (as a positive) regulators of the AGP1-CCR5 pathway (Sun et al., 2018; Wan et al., 2020).

Interestingly, *Orm1*-KO mice show altered metabolic parameters, such as increased levels of insulin and leptin together with impaired glucose tolerance (Sun et al., 2016) and AGP1 deficiency increases the expression of genes related to fibrosis in adipose tissue (Wang et al., 2021). Previously, and in agreement with the *Orm1*-KO mouse model, the continuous systemic infusion of hAGP1 improved glucose and insulin tolerance in obese/diabetic mice (Lee et al., 2010). Further explorations in the *Orm1*-KO mouse led to the discovery that AGP inhibits food intake. Mechanistically, AGP1 interacts with the leptin receptor (Lepr) in the hypothalamus and activates the JAK2-STAT3 pathway to inhibited food intake (Sun et al., 2016). However, it is not clear how AGP1, the main isoform in circulation, crosses the blood brain barrier to interact with the Lepr in the hypothalamus. Additionally, the main isoform in the brain is AGP2 and its levels do not change under metabolic stress (Sun et al., 2016). Even though, the nature of the AGP-Lepr interaction model is not completely understood, its existence is certainly an interesting observation. ApoD, the most ancestral Lipocalin in vertebrates, has also been shown to interact with the Lepr (Liu et al., 2001). ApoD interaction is thought to take place with the cytosolic domain of the Lepr (Liu et al., 2001), whereas AGP interaction was modeled to occur via the leptin-binding domain of the Lepr (Sun et al., 2016).

The first complete AGP-KO (*Orm1*, *Orm2* and *Orm3*-KO) was finally published last year, 2020 (Watanabe et al., 2020). The AGP-KO mice did not show any obvious defects in appearance or growth. However, the AGP-KO animals had exacerbated fibrosis, inflammatory response and macrophage infiltration in a model of renal fibrosis (Watanabe et al., 2020, 2021). Accordingly, AGP administration reduced renal fibrosis and inflammation (Bi et al., 2018). Interestingly, all-trans retinoic acid treatment boosted AGP serum concentration in plasma and required AGP to protect against renal fibrosis. So, how do all-trans retinoic acid and AGP damper renal fibrosis and the immune response? It is noteworthy that all-trans retinoic acid is a classical Lipocalin ligand and binds to AGP with micromolar affinity (Breustedt et al., 2006; Ruiz et al., 2013). Therefore, all-trans retinoic acid might just induce AGP expression that then transports it to the damaged area? Interestingly, the major transporter of retinol in plasma, the Lipocalin RBP4, is a negative acute-phase protein (Rosales et al., 1996). Thus, AGP could take the place of RBP4 and transport retinols during inflammation.

Interestingly, AGP1-KO did not affect the infarct area in a model of ischemic stroke (even when the blood brain barrier was compromised). Instead, the expression of AGP2 was induced in the ischemic tissue (Wan et al., 2016, 2019). Unfortunately, an AGP2-KO model was not available at that time. Therefore, the availability of a full AGP-KO is now a great tool to explore anew the role of AGP in the central nervous system. Expression of AGP2 in the brain is induced upon systemic inflammation, astrocytes being the main source of AGP. Mechanistically, AGP2 inhibited CCL4-induced microglial activation by blocking the interaction of AGP with CCR5 and reduced microglia-mediated neurotoxicity (Jo et al., 2017). Noteworthy, other Lipocalins are also expressed in glial cells. For instance, astrocytes express ApoD upon stress conditions to promote neuronal survival (Bajo-Graneras et al., 2011; Pascua-Maestro et al., 2018). Oppositely to AGP and ApoD, Lcn2 is an autocrine mediator of astrocytosis and renders astrocytes more sensitive to cell-death signals (Lee et al., 2009). To add one extra level of complexity, only apo-Lcn2 (no-ligand bound) sensitized activated astrocytes to cell-death (Lee et al., 2009). Therefore, it would be relevant to investigate if any ligand mediates the protection by AGP in the brain upon inflammation.

## Concluding Remarks

α_1_-acid glycoprotein expression is strongly up-regulated during the acute-phase response probably as a counter-balance to damper an excessive inflammatory response. Thus, AGP is typically associated with protection. Interestingly, AGP investigations are not limited to inflammation and new studies reported an active role of AGP in metabolic regulation. One of the most interesting features of AGP is its heterogeneity, from the amino acid sequence to the glycosylation pattern (Figure 1B). Multiple AGP forms are possible which suggests the existence of fine-tuned mechanisms to regulate AGP functions and highlights AGP versatility to participate in multiple process. The best example of AGP versatility is its ability to bind hundreds of small molecules (Figure 1B). Despite thousands of publications about AGP, its molecular functions are not fully understood. Hopefully, the newly created AGP-KO mice will help to shed light on AGP physiological roles.

## Author Contributions

The author confirms being the sole contributor of this work and has approved it for publication.

## Conflict of Interest

The author declares that the research was conducted in the absence of any commercial or financial relationships that could be construed as a potential conflict of interest.

## References

[B1] Ahnstrom J. Faber K. Axler O. Dahlback B. (2007). Hydrophobic ligand binding properties of the human lipocalin apolipoprotein M. *J Lipid Res* 48 1754–1762. 10.1194/jlr.m700103-jlr200 17525477

[B2] Albani J. R. (1997). Binding effect of progesterone on the dynamics of alpha1-acid glycoprotein. *Biochim Biophys Acta* 1336 349–359. 10.1016/s0304-4165(97)00043-3 9305808

[B3] Atemezem A. Mbemba E. Vassy R. Slimani H. Saffar L. Gattegno L. (2001). Human alpha1-acid glycoprotein binds to CCR5 expressed on the plasma membrane of human primary macrophages. *Biochem J* 356 121–128. 10.1042/0264-6021:3560121 11336643 PMC1221819

[B4] Baerenfaenger M. Meyer B. (2018). Intact Human Alpha-Acid Glycoprotein Analyzed by ESI-qTOF-MS: Simultaneous Determination of the Glycan Composition of Multiple Glycosylation Sites. *J Proteome Res* 17 3693–3703. 10.1021/acs.jproteome.8b00309 30295034

[B5] Bajo-Graneras R. Sanchez D. Gutierrez G. Gonzalez C. Do Carmo S. Rassart E. (2011). Apolipoprotein D alters the early transcriptional response to oxidative stress in the adult cerebellum. *J Neurochem* 117 949–960. 10.1111/j.1471-4159.2011.07266.x 21463325

[B6] Bi J. Watanabe H. Fujimura R. Nishida K. Nakamura R. Oshiro S. (2018). A downstream molecule of 1,25-dihydroxyvitamin D3, alpha-1-acid glycoprotein, protects against mouse model of renal fibrosis. *Sci Rep* 8 17329. 10.1038/s41598-018-35339-x PMC6255841 30478350

[B7] Breustedt D. A. Schonfeld D. L. Skerra A. (2006). Comparative ligand-binding analysis of ten human lipocalins. *Biochim Biophys Acta* 1764 161–173. 10.1016/j.bbapap.2005.12.006 16461020

[B8] Bteich M. (2019). An overview of albumin and alpha-1-acid glycoprotein main characteristics: highlighting the roles of amino acids in binding kinetics and molecular interactions. *Heliyon* 5 e02879. 10.1016/j.heliyon.2019.e02879 31844752 PMC6895661

[B9] Bteich M. Poulin P. Haddad S. (2021). Comparative Assessment of Extrapolation Methods Based on the Conventional Free Drug Hypothesis and Plasma Protein-Mediated Hepatic Uptake Theory for the Hepatic Clearance Predictions of Two Drugs Extensively Bound to Both the Albumin And Alpha-1-Acid Glycoprotein. *J Pharm Sci* 110 1385–1391. 10.1016/j.xphs.2020.11.009 33217427

[B10] Chiu K. M. Mortensen R. F. Osmand A. P. Gewurz H. (1977). Interactions of alpha1-acid glycoprotein with the immune system. I. Purification and effects upon lymphocyte responsiveness. *Immunology* 32 997–1005. 142068 PMC1445435

[B11] Christoffersen C. Obinata H. Kumaraswamy S. B. Galvani S. Ahnstrom J. Sevvana M. (2011). Endothelium-protective sphingosine-1-phosphate provided by HDL-associated apolipoprotein M. *Proc Natl Acad Sci U S A* 108 9613–9618. 10.1073/pnas.1103187108 21606363 PMC3111292

[B12] Costello M. Fiedel B. A. Gewurz H. (1979). Inhibition of platelet aggregation by native and desialised alpha-1 acid glycoprotein. *Nature* 281 677–678. 10.1038/281677a0 551286

[B13] Dassati S. Waldner A. Schweigreiter R. (2014). Apolipoprotein D takes center stage in the stress response of the aging and degenerative brain. *Neurobiol Aging* 35 1632–1642. 10.1016/j.neurobiolaging.2014.01.148 24612673 PMC3988949

[B14] De Graaf T. W. Van Der Stelt M. E. Anbergen M. G. Van Dijk W. (1993). Inflammation-induced expression of sialyl Lewis X-containing glycan structures on alpha 1-acid glycoprotein (orosomucoid) in human sera. *J Exp Med* 177 657–666. 10.1084/jem.177.3.657 7679706 PMC2190949

[B15] Dente L. Ruther U. Tripodi M. Wagner E. F. Cortese R. (1988). Expression of human alpha 1-acid glycoprotein genes in cultured cells and in transgenic mice. *Genes Dev* 2 259–266. 10.1101/gad.2.2.259 3360326

[B16] Dewey M. J. Rheaume C. Berger F. G. Baumann H. (1990). Inducible and tissue-specific expression of rat alpha-1-acid glycoprotein in transgenic mice. *J Immunol* 144 4392–4398. 2341724

[B17] di Masi A. Trezza V. Leboffe L. Ascenzi P. (2016). Human plasma lipocalins and serum albumin: Plasma alternative carriers? *J Control Release* 228 191–205. 10.1016/j.jconrel.2016.02.049 26951925

[B18] Ebihara T. Nishihara M. Takahashi J. Jinno F. Tagawa Y. (2018). Differences in nonclinical pharmacokinetics between species and prediction of human pharmacokinetics of TAK-272 (SCO-272), a novel orally active renin inhibitor. *Biopharm Drug Dispos* 39 175–183. 10.1002/bdd.2124 29474740

[B19] Ebihara T. Shimizu H. Yamamoto M. Higuchi T. Jinno F. Tagawa Y. (2019). The effect of elevated alpha1-acid glycoprotein on the pharmacokinetics of TAK-272 (SCO-272), an orally active renin inhibitor, in rats. *Xenobiotica* 49 584–590. 10.1080/00498254.2018.1480817 29790816

[B20] Eichinger A. Nasreen A. Kim H. J. Skerra A. (2007). Structural insight into the dual ligand specificity and mode of high density lipoprotein association of apolipoprotein D. *J Biol Chem* 282 31068–31075. 10.1074/jbc.m703552200 17699160

[B21] Fernandes C. L. Ligabue-Braun R. Verli H. (2015). Structural glycobiology of human alpha1-acid glycoprotein and its implications for pharmacokinetics and inflammation. *Glycobiology* 25 1125–1133. 10.1093/glycob/cwv041 26088564

[B22] Flower D. R. North A. C. Sansom C. E. (2000). The lipocalin protein family: structural and sequence overview. *Biochim Biophys Acta* 1482 9–24. 10.1016/s0167-4838(00)00148-5 11058743

[B23] Fluckinger M. Haas H. Merschak P. Glasgow B. J. Redl B. (2004). Human tear lipocalin exhibits antimicrobial activity by scavenging microbial siderophores. *Antimicrob Agents Chemother* 48 3367–3372. 10.1128/aac.48.9.3367-3372.2004 15328098 PMC514737

[B24] Fournier T. Medjoubi N. N. Porquet D. (2000). Alpha-1-acid glycoprotein. *Biochim Biophys Acta* 1482 157–171. 11058758 10.1016/s0167-4838(00)00153-9

[B25] Frej C. Mendez A. J. Ruiz M. Castillo M. Hughes T. A. Dahlback B. (2017). A Shift in ApoM/S1P Between HDL-Particles in Women With Type 1 Diabetes Mellitus Is Associated With Impaired Anti-Inflammatory Effects of the ApoM/S1P Complex. *Arterioscler Thromb Vasc Biol* 37 1194–1205. 10.1161/atvbaha.117.309275 28385702

[B26] Fuse E. Tanii H. Kurata N. Kobayashi H. Shimada Y. Tamura T. (1998). Unpredicted clinical pharmacology of UCN-01 caused by specific binding to human alpha1-acid glycoprotein. *Cancer Res* 58 3248–3253. 9699650

[B27] Fuse E. Tanii H. Takai K. Asanome K. Kurata N. Kobayashi H. (1999). Altered pharmacokinetics of a novel anticancer drug, UCN-01, caused by specific high affinity binding to alpha1-acid glycoprotein in humans. *Cancer Res* 59 1054–1060. 10070963

[B28] Ganfornina M. D. Gutierrez G. Bastiani M. Sanchez D. (2000). A phylogenetic analysis of the lipocalin protein family. *Mol Biol Evol* 17 114–126. 10.1093/oxfordjournals.molbev.a026224 10666711

[B29] Ganguly M. Carnighan R. H. Westphal U. (1967). Steroid-protein interactions. XIV. Interaction between human alpha 1-acid glycoprotein and progesterone. *Biochemistry* 6 2803–2814. 10.1021/bi00861a022 6055192

[B30] Gannon B. M. Glesby M. J. Finkelstein J. L. Raj T. Erickson D. Mehta S. (2019). A point-of-care assay for alpha-1-acid glycoprotein as a diagnostic tool for rapid, mobile-based determination of inflammation. *Curr Res Biotechnol* 1 41–48. 10.1016/j.crbiot.2019.09.002 32342042 PMC7185229

[B31] Gasymov O. K. Abduragimov A. R. Yusifov T. N. Glasgow B. J. (1998). Structural changes in human tear lipocalins associated with lipid binding. *Biochim Biophys Acta* 1386 145–156. 10.1016/s0167-4838(98)00092-2 9675263

[B32] Gunnarsson P. Fornander L. Pahlsson P. Grenegard M. (2010). Sialic acid residues play a pivotal role in alpha(1)-acid glycoprotein (AGP)-induced generation of reactive oxygen species in chemotactic peptide pre-activated neutrophil granulocytes. *Inflamm Res* 59 89–95. 10.1007/s00011-009-0071-1 19669698

[B33] Gunnarsson P. Levander L. Pahlsson P. Grenegard M. (2007). The acute-phase protein alpha 1-acid glycoprotein (AGP) induces rises in cytosolic Ca2+ in neutrophil granulocytes via sialic acid binding immunoglobulin-like lectins (siglecs). *FASEB J* 21 4059–4069. 10.1096/fj.07-8534com 17675532

[B34] Gutierrez G. Ganfornina M. D. Sanchez D. (2000). Evolution of the lipocalin family as inferred from a protein sequence phylogeny. *Biochim Biophys Acta* 1482 35–45. 10.1016/s0167-4838(00)00151-5 11058745

[B35] Hanada K. (2017). Lipophilicity Influences Drug Binding to alpha1-Acid Glycoprotein F1/S Variants But Not to the A Variant. *Drugs R D* 17 475–480. 10.1007/s40268-017-0193-9 28646384 PMC5629133

[B36] Haraldsson B. S. Johnsson E. K. Rippe B. (1992). Glomerular permselectivity is dependent on adequate serum concentrations of orosomucoid. *Kidney Int* 41 310–316. 10.1038/ki.1992.43 1552704

[B37] Havenaar E. C. Hoff R. C. Van Den Eijnden D. H. Van Dijk W. (1998). Sialyl Lewis(x) epitopes do not occur on acute phase proteins in mice: relationship to the absence of alpha3-fucosyltransferase in the liver. *Glycoconj J* 15 389–395. 9613826 10.1023/a:1006977903048

[B38] Herve F. Caron G. Duche J. C. Gaillard P. Abd Rahman N. Tsantili-Kakoulidou A. (1998). Ligand specificity of the genetic variants of human alpha1-acid glycoprotein: generation of a three-dimensional quantitative structure-activity relationship model for drug binding to the A variant. *Mol Pharmacol* 54 129–138. 10.1124/mol.54.1.129 9658198

[B39] Herve F. Duche J. C. D’athis P. Marche C. Barre J. Tillement J. P. (1996). Binding of disopyramide, methadone, dipyridamole, chlorpromazine, lignocaine and progesterone to the two main genetic variants of human alpha 1-acid glycoprotein: evidence for drug-binding differences between the variants and for the presence of two separate drug-binding sites on alpha 1-acid glycoprotein. *Pharmacogenetics* 6 403–415. 10.1097/00008571-199610000-00004 8946472

[B40] Herve F. Gomas E. Duche J. C. Tillement J. P. (1993). Evidence for differences in the binding of drugs to the two main genetic variants of human alpha 1-acid glycoprotein. *Br J Clin Pharmacol* 36 241–249. 10.1111/j.1365-2125.1993.tb04224.x 9114911 PMC1364645

[B41] Hochepied T. Berger F. G. Baumann H. Libert C. (2003). Alpha(1)-acid glycoprotein: an acute phase protein with inflammatory and immunomodulating properties. *Cytokine Growth Factor Rev* 14 25–34. 10.1016/s1359-6101(02)00054-0 12485617

[B42] Hochepied T. Van Molle W. Berger F. G. Baumann H. Libert C. (2000). Involvement of the acute phase protein alpha 1-acid glycoprotein in nonspecific resistance to a lethal gram-negative infection. *J Biol Chem* 275 14903–14909. 10.1074/jbc.275.20.14903 10809735

[B43] Hochepied T. Wullaert A. Berger F. G. Baumann H. Brouckaert P. Steidler L. (2002). Overexpression of alpha(1)-acid glycoprotein in transgenic mice leads to sensitisation to acute colitis. *Gut* 51 398–404. 10.1136/gut.51.3.398 12171963 PMC1773348

[B44] Huang F. Subbaiah P. V. Holian O. Zhang J. Johnson A. Gertzberg N. (2005). Lysophosphatidylcholine increases endothelial permeability: role of PKCalpha and RhoA cross talk. *Am J Physiol Lung Cell Mol Physiol* 289 L176–L185. 15764646 10.1152/ajplung.00003.2005

[B45] Huang R. Y. Hudgens J. W. (2013). Effects of desialylation on human alpha1-acid glycoprotein-ligand interactions. *Biochemistry* 52 7127–7136. 10.1021/bi4011094 24041412

[B46] Hudry-Clergeon H. Stengel D. Ninio E. Vilgrain I. (2005). Platelet-activating factor increases VE-cadherin tyrosine phosphorylation in mouse endothelial cells and its association with the PtdIns3’-kinase. *FASEB J* 19 512–520. 15791001 10.1096/fj.04-2202com PMC4848345

[B47] Israili Z. H. Dayton P. G. (2001). Human alpha-1-glycoprotein and its interactions with drugs. *Drug Metab Rev* 33 161–235. 10.1081/dmr-100104402 11495502

[B48] Jo M. Kim J. H. Song G. J. Seo M. Hwang E. M. Suk K. (2017). Astrocytic Orosomucoid-2 Modulates Microglial Activation and Neuroinflammation. *J Neurosci* 37 2878–2894. 10.1523/jneurosci.2534-16.2017 28193696 PMC6596722

[B49] Johnsson E. Haraldsson B. (1993). Addition of purified orosomucoid preserves the glomerular permeability for albumin in isolated perfused rat kidneys. *Acta Physiol Scand* 147 1–8. 10.1111/j.1748-1716.1993.tb09466.x 8452035

[B50] Jorgensen H. G. Elliott M. A. Priest R. Smith K. D. (1998). Modulation of sialyl Lewis X dependent binding to E-selectin by glycoforms of alpha-1-acid glycoprotein expressed in rheumatoid arthritis. *Biomed Chromatogr* 12 343–349. 10.1002/(sici)1099-0801(199811/12)12:6<343::aid-bmc760>3.0.co;2-6 9861495

[B51] Keser T. Tijardovic M. Gornik I. Lukic E. Lauc G. Gornik O. (2021). High-throughput and site-specific N-glycosylation analysis of human alpha-1-acid glycoprotein offers a great potential for new biomarker discovery. *Mol Cell Proteomics* 20 100044. 10.1074/mcp.ra120.002433 33493676 PMC7950198

[B52] Kindberg G. M. Gudmundsen O. Berg T. (1990). The effect of vanadate on receptor-mediated endocytosis of asialoorosomucoid in rat liver parenchymal cells. *J Biol Chem* 265 8999–9005. 10.1016/s0021-9258(19)38802-7 2345164

[B53] Kono K. Fukuchi Y. Okawa H. Nunoya K. I. Imawaka H. Watanabe H. (2019). Unique Hydrolysis of an Ester-Type Prodrug of Levodopa in Human Plasma: Relay-Type Role Sharing between Alpha-1 Acid Glycoprotein and Human Serum Albumin. *Mol Pharm* 16 4131–4138. 10.1021/acs.molpharmaceut.9b00435 31433646

[B54] Kono K. Nunoya K. I. Nakamura Y. Bi J. Mukunoki A. Takeo T. (2021). Species Difference in Hydrolysis of an Ester-type Prodrug of Levodopa in Human and Animal Plasma: Different Contributions of Alpha-1 Acid Glycoprotein. *Mol Pharm* 18 1985–1991. 10.1021/acs.molpharmaceut.0c01134 33861617

[B55] Kremer J. M. Wilting J. Janssen L. H. (1988). Drug binding to human alpha-1-acid glycoprotein in health and disease. *Pharmacol Rev* 40 1–47. 3064105

[B56] Kuroda Y. Matsumoto S. Shibukawa A. Nakagawa T. (2003). Capillary electrophoretic study on pH dependence of enantioselective disopyramide binding to genetic variants of human alpha1-acid glycoprotein. *Analyst* 128 1023–1027. 10.1039/b212850k 12964601

[B57] Lee S. Park J. Y. Lee W. H. Kim H. Park H. C. Mori K. (2009). Lipocalin-2 is an autocrine mediator of reactive astrocytosis. *J Neurosci* 29 234–249. 10.1523/jneurosci.5273-08.2009 19129400 PMC6664907

[B58] Lee S. H. Choi J. M. Jung S. Y. Cox A. R. Hartig S. M. Moore D. D. (2021). The bile acid induced hepatokine orosomucoid suppresses adipocyte differentiation. *Biochem Biophys Res Commun* 534 864–870. 10.1016/j.bbrc.2020.10.086 33168190 PMC7785631

[B59] Lee Y. S. Choi J. W. Hwang I. Lee J. W. Lee J. H. Kim A. Y. (2010). Adipocytokine orosomucoid integrates inflammatory and metabolic signals to preserve energy homeostasis by resolving immoderate inflammation. *J Biol Chem* 285 22174–22185. 10.1074/jbc.m109.085464 20442402 PMC2903347

[B60] Lei H. Sun Y. Luo Z. Yourek G. Gui H. Yang Y. (2016). Fatigue-induced Orosomucoid 1 Acts on C-C Chemokine Receptor Type 5 to Enhance Muscle Endurance. *Sci Rep* 6 18839. 10.1038/srep18839 PMC4703980 26740279

[B61] Liang J. Zhu J. Wang M. Singal A. G. Odewole M. Kagan S. (2019). Evaluation of AGP Fucosylation as a Marker for Hepatocellular Carcinoma of Three Different Etiologies. *Sci Rep* 9 11580. 10.1038/s41598-019-48043-1 PMC6689003 31399619

[B62] Libert C. Brouckaert P. Fiers W. (1994). Protection by alpha 1-acid glycoprotein against tumor necrosis factor-induced lethality. *J Exp Med* 180 1571–1575. 10.1084/jem.180.4.1571 7931089 PMC2191695

[B63] Liu Z. Chang G. Q. Leibowitz S. F. (2001). Apolipoprotein D interacts with the long-form leptin receptor: a hypothalamic function in the control of energy homeostasis. *FASEB J* 15 1329–1331. 10.1096/fj.00-0530fje 11344130

[B64] Logdberg L. Wester L. (2000). Immunocalins: a lipocalin subfamily that modulates immune and inflammatory responses. *Biochim Biophys Acta* 1482 284–297. 10.1016/s0167-4838(00)00164-3 11058769

[B65] Mathiesen Janiurek M. Soylu-Kucharz R. Christoffersen C. Kucharz K. Lauritzen M. (2019). Apolipoprotein M-bound sphingosine-1-phosphate regulates blood-brain barrier paracellular permeability and transcytosis. *Elife* 8 e49405. 10.7554/eLife.49405 PMC6877292 31763978

[B66] Matsumoto K. Nishi K. Kikuchi M. Watanabe H. Nakajou K. Komori H. (2010). Receptor-mediated uptake of human alpha1-acid glycoprotein into liver parenchymal cells in mice. *Drug Metab Pharmacokinet* 25 101–107. 10.2133/dmpk.25.101 20208393

[B67] McDonald C. M. Suchdev P. S. Krebs N. F. Hess S. Y. Wessells K. R. Ismaily S. (2020). Adjusting plasma or serum zinc concentrations for inflammation: Biomarkers Reflecting Inflammation and Nutritional Determinants of Anemia (BRINDA) project. *Am J Clin Nutr* 111 927–937. 10.1093/ajcn/nqz304 32266402 PMC7138668

[B68] McNamara P. J. Brouwer K. R. Gillespie M. N. (1986). Autacoid binding to serum proteins. Interaction of platelet activating factor (PAF) with human serum alpha-1-acid glycoprotein (AAG). *Biochem Pharmacol* 35 621–624. 3947392 10.1016/0006-2952(86)90357-6

[B69] Mic M. Pirnau A. Floare C. G. Bogdan M. (2020). Study of the binding affinity between imatinib and alpha-1 glycoprotein using nuclear spin relaxation and isothermal titration calorimetry. *Int J Biol Macromol* 147 326–332. 10.1016/j.ijbiomac.2020.01.077 31951849

[B70] Moore D. F. Rosenfeld M. R. Gribbon P. M. Winlove C. P. Tsai C. M. (1997). Alpha-1-acid (AAG, orosomucoid) glycoprotein: interaction with bacterial lipopolysaccharide and protection from sepsis. *Inflammation* 21 69–82. 9179623 10.1023/a:1027342909423

[B71] Muchitsch E. M. Auer W. Pichler L. (1998). Effects of alpha 1-acid glycoprotein in different rodent models of shock. *Fundam Clin Pharmacol* 12 173–181. 10.1111/j.1472-8206.1998.tb00938.x 9565771

[B72] Muchitsch E. M. Teschner W. Linnau Y. Pichler L. (1996). In vivo effect of alpha 1-acid glycoprotein on experimentally enhanced capillary permeability in guinea-pig skin. *Arch Int Pharmacodyn Ther* 331 313–321. 9125002

[B73] Muchitsch E. M. Varadi K. Pichler L. (2000). Effects of alpha 1-acid glycoprotein on acute pancreatitis and acute lung injury in rats. *Arzneimittelforschung* 50 987–994. 10.1055/s-0031-1300322 11148865

[B74] Nishi K. Komine Y. Fukunaga N. Maruyama T. Suenaga A. Otagiri M. (2006). Involvement of disulfide bonds and histidine 172 in a unique beta-sheet to alpha-helix transition of alpha 1-acid glycoprotein at the biomembrane interface. *Proteins* 63 611–620. 10.1002/prot.20923 16470806

[B75] Nishi K. Maruyama T. Halsall H. B. Handa T. Otagiri M. (2004). Binding of alpha1-acid glycoprotein to membrane results in a unique structural change and ligand release. *Biochemistry* 43 10513–10519. 10.1021/bi0400204 15301549

[B76] Nishi K. Ono T. Nakamura T. Fukunaga N. Izumi M. Watanabe H. (2011). Structural insights into differences in drug-binding selectivity between two forms of human alpha1-acid glycoprotein genetic variants, the A and F1^∗^S forms. *J Biol Chem* 286 14427–14434. 10.1074/jbc.m110.208926 21349832 PMC3077642

[B77] Nishi K. Sakai N. Komine Y. Maruyama T. Halsall H. B. Otagiri M. (2002). Structural and drug-binding properties of alpha(1)-acid glycoprotein in reverse micelles. *Biochim Biophys Acta* 1601 185–191. 10.1016/s1570-9639(02)00465-x 12445481

[B78] Nishi K. Sakurama K. Kobashigawa Y. Morioka H. Udo N. Hashimoto M. (2019). Interaction of Aripiprazole With Human alpha1-Acid Glycoprotein. *J Pharm Sci* 108 3911–3916. 31520646 10.1016/j.xphs.2019.09.003

[B79] Ojala P. J. Hermansson M. Tolvanen M. Polvinen K. Hirvonen T. Impola U. (2006). Identification of alpha-1 acid glycoprotein as a lysophospholipid binding protein: a complementary role to albumin in the scavenging of lysophosphatidylcholine. *Biochemistry* 45 14021–14031. 10.1021/bi061657l 17115697

[B80] Ongay S. Neususs C. (2010). Isoform differentiation of intact AGP from human serum by capillary electrophoresis-mass spectrometry. *Anal Bioanal Chem* 398 845–855. 10.1007/s00216-010-3948-5 20617306

[B81] Pascua-Maestro R. Gonzalez E. Lillo C. Ganfornina M. D. Falcon-Perez J. M. Sanchez D. (2018). Extracellular Vesicles Secreted by Astroglial Cells Transport Apolipoprotein D to Neurons and Mediate Neuronal Survival Upon Oxidative Stress. *Front Cell Neurosci* 12:526. 10.3389/fncel.2018.00526 PMC6335244 30687015

[B82] Pearlman W. H. Gueriguian J. L. Sawyer M. E. (1973). A specific progesterone-binding component of human breast cyst fluid. *J Biol Chem* 248 5736–5741. 10.1016/s0021-9258(19)43566-7 4723913

[B83] Qin X. Y. Hara M. Arner E. Kawaguchi Y. Inoue I. Tatsukawa H. (2017). Transcriptome Analysis Uncovers a Growth-Promoting Activity of Orosomucoid-1 on Hepatocytes. *EBioMedicine* 24 257–266. 10.1016/j.ebiom.2017.09.008 28927749 PMC5652006

[B84] Qin Z. Wan J. J. Sun Y. Wang P. Y. Su D. F. Lei H. (2016). ORM Promotes Skeletal Muscle Glycogen Accumulation via CCR5-Activated AMPK Pathway in Mice. *Front Pharmacol* 7:302. 10.3389/fphar.2016.00302 PMC5020064 27679573

[B85] Rassart E. Bedirian A. Do Carmo S. Guinard O. Sirois J. Terrisse L. (2000). Apolipoprotein D. *Biochim Biophys Acta* 1482 185–198. 11058760 10.1016/s0167-4838(00)00162-x

[B86] Rosales F. J. Ritter S. J. Zolfaghari R. Smith J. E. Ross A. C. (1996). Effects of acute inflammation on plasma retinol, retinol-binding protein, and its mRNA in the liver and kidneys of vitamin A-sufficient rats. *J Lipid Res* 37 962–971. 10.1016/s0022-2275(20)42007-3 8725149

[B87] Rose A. S. Bradley A. R. Valasatava Y. Duarte J. M. Prlic A. Rose P. W. (2018). NGL viewer: web-based molecular graphics for large complexes. *Bioinformatics* 34 3755–3758. 10.1093/bioinformatics/bty419 29850778 PMC6198858

[B88] Ruiz M. Frej C. Holmer A. Guo L. J. Tran S. Dahlback B. (2017). High-Density Lipoprotein-Associated Apolipoprotein M Limits Endothelial Inflammation by Delivering Sphingosine-1-Phosphate to the Sphingosine-1-Phosphate Receptor 1. *Arterioscler Thromb Vasc Biol* 37 118–129. 10.1161/atvbaha.116.308435 27879252

[B89] Ruiz M. Sanchez D. Correnti C. Strong R. K. Ganfornina M. D. (2013). Lipid-binding properties of human ApoD and Lazarillo-related lipocalins: functional implications for cell differentiation. *FEBS J* 280 3928–3943. 10.1111/febs.12394 23777559

[B90] Sager G. Bratlid H. Little C. (1987). Binding of catecholamines to alpha-1 acid glycoprotein, albumin and lipoproteins in human serum. *Biochem Pharmacol* 36 3607–3612. 10.1016/0006-2952(87)90009-8 3675618

[B91] Samsonov S. A. Zsila F. Maszota-Zieleniak M. (2021). Acute phase alpha1-acid glycoprotein as a siderophore-capturing component of the human plasma: A molecular modeling study. *J Mol Graph Model* 105 107861. 10.1016/j.jmgm.2021.107861 33640788

[B92] Sanchez D. Ganfornina M. D. Gutierrez G. Marin A. (2003). Exon-intron structure and evolution of the Lipocalin gene family. *Mol Biol Evol* 20 775–783. 10.1093/molbev/msg079 12679526

[B93] Schiefner A. Skerra A. (2015). The menagerie of human lipocalins: a natural protein scaffold for molecular recognition of physiological compounds. *Acc Chem Res* 48 976–985. 10.1021/ar5003973 25756749

[B94] Schmid K. (1950). Preparation and Properties of an Acid Glycoprotein Prepared from Human Plasma. *J. Am. Chem. Soc* 72 2816. 10.1021/ja01162a553

[B95] Schonfeld D. L. Ravelli R. B. Mueller U. Skerra A. (2008). The 1.8-A crystal structure of alpha1-acid glycoprotein (Orosomucoid) solved by UV RIP reveals the broad drug-binding activity of this human plasma lipocalin. *J Mol Biol* 384 393–405. 10.1016/j.jmb.2008.09.020 18823996

[B96] Seddiki N. Rabehi L. Benjouad A. Saffar L. Ferriere F. Gluckman J. C. (1997). Effect of mannosylated derivatives on HIV-1 infection of macrophages and lymphocytes. *Glycobiology* 7 1229–1236. 10.1093/glycob/7.8.1229 9455924

[B97] Shemyakin I. G. Pukhalsky A. L. Stepanshina V. N. Shmarina G. V. Aleshkin V. A. Afanas’ev S. S. (2005). Preventive and therapeutic effects of alpha-acid glycoprotein in mice infected with B. anthracis. *Bull Exp Biol Med* 140 439–444. 10.1007/s10517-005-0514-9 16671576

[B98] Shi M. Ma X. Yang Q. Wang W. Li X. Song X. (2021). miR-362-3p Targets Orosomucoid 1 to Promote Cell Proliferation, Restrain Cell Apoptosis and Thereby Mitigate Hypoxia/Reoxygenation-Induced Cardiomyocytes Injury. *Cardiovasc Toxicol* 21 387–398. 10.1007/s12012-020-09631-0 33459949

[B99] Smith S. A. Gagnon S. Waters N. J. (2017). Mechanistic investigations into the species differences in pinometostat clearance: impact of binding to alpha-1-acid glycoprotein and permeability-limited hepatic uptake. *Xenobiotica* 47 185–193. 10.3109/00498254.2016.1173265 27160567

[B100] Smith S. A. Waters N. J. (2018). Pharmacokinetic and Pharmacodynamic Considerations for Drugs Binding to Alpha-1-Acid Glycoprotein. *Pharm Res* 36 30. 10.1007/s11095-018-2551-x PMC7089466 30593605

[B101] Sorensson J. Matejka G. L. Ohlson M. Haraldsson B. (1999). Human endothelial cells produce orosomucoid, an important component of the capillary barrier. *Am J Physiol* 276 H530–H534. 9950854 10.1152/ajpheart.1999.276.2.H530

[B102] Sun Y. Qin Z. Wan J. J. Wang P. Y. Yang Y. L. Yu J. G. (2018). Estrogen weakens muscle endurance via estrogen receptor-p38 MAPK-mediated orosomucoid (ORM) suppression. *Exp Mol Med* 50 e463. 10.1038/emm.2017.307 29869624 PMC5898901

[B103] Sun Y. Yang Y. Qin Z. Cai J. Guo X. Tang Y. (2016). The Acute-Phase Protein Orosomucoid Regulates Food Intake and Energy Homeostasis via Leptin Receptor Signaling Pathway. *Diabetes* 65 1630–1641. 10.2337/db15-1193 27207522

[B104] Taguchi K. Nishi K. Giam Chuang V. T. Maruyama T. Otagiri M. (2013). “Molecular Aspects of Human Alpha-1 Acid Glycoprotein — Structure and Function,” in *Acute Phase Proteins*, ed. Janciauskiene S. 139–162.

[B105] Treuheit M. J. Costello C. E. Halsall H. B. (1992). Analysis of the five glycosylation sites of human alpha 1-acid glycoprotein. *Biochem J* 283(Pt 1), 105–112. 10.1042/bj2830105 1567356 PMC1131000

[B106] Wan J. Qin Z. Lei H. Wang P. Zhang Y. Feng J. (2020). Erythromycin has therapeutic efficacy on muscle fatigue acting specifically on orosomucoid to increase muscle bioenergetics and physiological parameters of endurance. *Pharmacol Res* 161 105118. 10.1016/j.phrs.2020.105118 32777256

[B107] Wan J. J. Qin Z. Liu X. (2016). ORM Elevation in Response to Cognitive Impairment Is an Accompanying Phenomenon. *CNS Neurosci Ther* 22 723–724. 10.1111/cns.12586 27390178 PMC6492861

[B108] Wan J. J. Wang P. Y. Zhang Y. Qin Z. Sun Y. Hu B. H. (2019). Role of acute-phase protein ORM in a mice model of ischemic stroke. *J Cell Physiol* 234 20533–20545. 10.1002/jcp.28653 31026065

[B109] Wang B. L. Kou S. B. Lin Z. Y. Shi J. H. Liu Y. X. (2020). Insights on the interaction mechanism of brigatinib to human alpha-1-acid glycoprotein: Experimental and computational approaches. *Int J Biol Macromol* 157 340–349. 10.1016/j.ijbiomac.2020.04.151 32335105

[B110] Wang P. Y. Feng J. Y. Zhang Z. Chen Y. Qin Z. Dai X. M. (2021). The adipokine orosomucoid alleviates adipose tissue fibrosis via the AMPK pathway. *Acta Pharmacol Sin* 33875797. 10.1038/s41401-021-00666-9 PMC8792011 33875797

[B111] Watanabe H. Bi J. Murata R. Fujimura R. Nishida K. Imafuku T. (2020). A synthetic retinoic acid receptor agonist Am80 ameliorates renal fibrosis via inducing the production of alpha-1-acid glycoprotein. *Sci Rep* 10 11424. 10.1038/s41598-020-68337-z PMC7351735 32651445

[B112] Watanabe H. Fujimura R. Hiramoto Y. Murata R. Nishida K. Bi J. (2021). An acute phase protein alpha1-acid glycoprotein mitigates AKI and its progression to CKD through its anti-inflammatory action. *Sci Rep* 11 7953. 10.1038/s41598-021-87217-8 PMC8041882 33846468

[B113] Weimer H. E. Mehl J. W. Winzler R. J. (1950). Studies on the mucoproteins of human plasma. V. Isolation and characterization of a homogeneous mucoprotein. *J Biol Chem* 185 561–568. 14774397

[B114] Westphal U. Ashley B. D. Selden G. L. (1961). Steroid-protein interactions. VII. Interactions of progesterone and corticosteroids with human plasma proteins determined by multiple equilibrium dialysis. *Arch Biochem Biophys* 92 441–448. 13784641 10.1016/0003-9861(61)90383-6

[B115] Wishart D. S. Knox C. Guo A. C. Shrivastava S. Hassanali M. Stothard P. (2006). DrugBank: a comprehensive resource for in silico drug discovery and exploration. *Nucleic Acids Res* 34 D668–D672. 16381955 10.1093/nar/gkj067 PMC1347430

[B116] Wu D. Struwe W. B. Harvey D. J. Ferguson M. A. J. Robinson C. V. (2018). N-glycan microheterogeneity regulates interactions of plasma proteins. *Proc Natl Acad Sci U S A* 115 8763–8768. 10.1073/pnas.1807439115 30111543 PMC6126716

[B117] Xu N. Zhang X. Y. Dong X. Ekstrom U. Ye Q. Nilsson-Ehle P. (2002). Effects of platelet-activating factor, tumor necrosis factor, and interleukin-1alpha on the expression of apolipoprotein M in HepG2 cells. *Biochem Biophys Res Commun* 292 944–950. 10.1006/bbrc.2002.6755 11944906

[B118] Yamauchi M. Kusano H. Nakakura M. Kato Y. (2005). Reducing the impact of binding of UCN-01 to human alpha1-acid glycoprotein by encapsulation in liposomes. *Biol Pharm Bull* 28 1259–1264. 10.1248/bpb.28.1259 15997110

[B119] Yamauchi M. Kusano H. Saito E. Abe M. Tsutsumi K. Uosaki Y. (2008). Controlled release of a protein kinase inhibitor UCN-01 from liposomes influenced by the particle size. *Int J Pharm* 351 250–258. 10.1016/j.ijpharm.2007.08.021 17904317

[B120] Yuan Z. Q. Qiao C. Yang Z. C. Yu L. Sun L. N. Qian Y. (2020). The Impact of Plasma Protein Binding Characteristics and Unbound Concentration of Voriconazole on Its Adverse Drug Reactions. *Front Pharmacol* 11:505. 10.3389/fphar.2020.00505 PMC7194128 32390847

[B121] Zsila F. Mady G. (2008). Biliverdin is the endogenous ligand of human serum alpha1-acid glycoprotein. *Biochem Biophys Res Commun* 372 503–507. 10.1016/j.bbrc.2008.05.090 18510947

[B122] Zsila F. Matsunaga H. Bikadi Z. Haginaka J. (2006). Multiple ligand-binding properties of the lipocalin member chicken alpha1-acid glycoprotein studied by circular dichroism and electronic absorption spectroscopy: the essential role of the conserved tryptophan residue. *Biochim Biophys Acta* 1760 1248–1273. 10.1016/j.bbagen.2006.04.006 16813999

[B123] Zsila F. Visy J. Mady G. Fitos I. (2008). Selective plasma protein binding of antimalarial drugs to alpha1-acid glycoprotein. *Bioorg Med Chem* 16 3759–3772. 10.1016/j.bmc.2008.01.053 18289858

